# Transcriptomic Association of COL3A1^+^ Fibroblasts With Mechanical Pain–Related Gene Signatures in Triple‐Negative Breast Cancer

**DOI:** 10.1155/prm/8480126

**Published:** 2026-08-03

**Authors:** Yuting Zhong, Qi Sun, Changgang Sun

**Affiliations:** ^1^ School of Integrative Medicine, Shanghai University of Traditional Chinese Medicine, Shanghai 201203, China, shutcm.edu.cn; ^2^ College of First Clinical Medicine, Shandong University of Traditional Chinese Medicine, Jinan Shandong, 250000, China, sdutcm.edu.cn; ^3^ College of Traditional Chinese Medicine, Shandong Second Medical University, Weifang Shandong, 261000, China; ^4^ Department of Oncology, Weifang Traditional Chinese Hospital, Weifang Shandong, 261000, China, wfszyy.com

**Keywords:** COL3A1, mechanotransduction, pain, single-cell RNA sequencing, triple-negative breast cancer

## Abstract

**Background:**

Epidemiological data show that approximately 80% of cancer patients experience pain of varying degrees throughout the course of their disease, with nearly one‐third experiencing severe pain, significantly impacting their quality of life and the effectiveness of antitumor treatment. Triple‐negative (TN) breast cancer tissues typically exhibit increased stromal stiffness and abnormally elevated mechanical stress; these biomechanical alterations may amplify pain signals by activating mechanosensitive channels. Utilizing single‐cell RNA sequencing analysis, this study aims to elucidate the potential biological links between fibroblast mechanotransduction and cancer‐associated pain, thereby providing a theoretical basis for clinical diagnosis and treatment.

**Methods:**

Dimensionality reduction and unsupervised clustering were used to identify cell types in TN breast cancer single‐cell RNA sequencing data. To assess the association between pain and mechanical stimulation, we constructed a set of gene signatures associated with mechanical stimulation and pain and calculated scores using the Area Under the Curve Cell (AUCell). CellChat and SCENIC were used to reveal the communication networks and transcription factor regulatory mechanisms of fibroblast subtypes.

**Results:**

COL3A1^+^ fibroblasts derived from TN breast cancer are highly involved in biological processes such as extracellular matrix remodeling, collagen fiber formation, and mechanotransduction. To assess the association between pain and mechanical stimulation, we constructed a gene signature set related to mechanical stimuli and pain and calculated corresponding scores using the AUCell tool. Cell communication studies showed that COL3A1^+^ fibroblasts interact extensively with epithelial cells and other cells through laminin and collagen signaling pathways, potentially leading to mechanotransduction remodeling of the TN breast cancer microenvironment.

**Conclusion:**

COL3A1^+^ fibroblasts demonstrate enhanced transcriptional profiles pertinent to collagen deposition and cytoskeletal reorganization, which are correlated with mechanotransduction signaling and may be connected with mechanical sensitivity in cancer‐related pain. This study systematically characterizes the potential relationship between fibroblast‐associated mechanotransduction characteristics and pain‐related gene signatures at the single‐cell level in TN breast cancer. These findings offer hypothesis‐generating insights into the molecular landscape of tumor‐associated pain, although additional experimental and clinical validation is necessary.

## 1. Introduction

Breast cancer is one of the most common and biologically diverse malignant tumors worldwide [1], accounting for a large proportion of cancer diagnoses and deaths among women [2]. Despite decades of research and advancements in screening and treatment [3, 4], breast cancer remained the second leading cause of cancer‐related deaths and the fourth leading cause of cancer death [5]. The global burden of breast cancer is projected to increase significantly by 2040, with more than 3 million new cases and 1 million deaths annually [6, 7].

Triple‐negative (TN) breast cancer, the most aggressive and poorest‐prognostic subtype of breast cancer, has a high incidence of pain, placing a heavy pathophysiological burden on patients [8–10]. As one of the most common symptoms, pain can be caused by local tumor compression and infiltration, or by nerve damage and tissue changes resulting from surgery, radiotherapy, or chemotherapy [11, 12]. This pain is usually chronic and long‐lasting, and its intensity may fluctuate and worsen with disease progression or treatment interventions, severely interfering with patients′ daily lives and sleep. More importantly, long‐term pain stimulation not only leads to limited physiological function but is also often accompanied by mood disorders such as anxiety and depression, forming a vicious cycle of mind–body interaction, significantly reducing patients’ quality of life, and adversely affecting overall prognosis and recovery [12, 13]. In clinical practice, the degree of pain is not entirely consistent with tumor size or extent of invasion [14]. This suggests that the mechanism of pain development is not solely determined by tumor burden but may involve more complex microenvironmental regulatory factors [15].

From the perspective of pain physiology, mechanical stimulation is one of the important triggers of pain. Increased tissue stiffness or abnormally elevated mechanical stress can directly activate mechanosensitive ion channels located on cell membranes, thereby initiating the transmission process of pain signals [16, 17]. This process involves converting physical forces into intracellular electrochemical signals, triggering neuronal excitation through transmembrane ion flow [18]. Studies have shown that mechanosensitive ion channels undergo conformational changes upon sensing tension changes, leading to channel opening and the influx of cations (such as sodium and calcium ions) down their concentration gradient, thereby causing cell membrane depolarization. When the depolarization reaches the threshold potential level, it triggers the generation of an action potential, ultimately converting the mechanical stimulus signal into a transmissible electrical signal in the nervous system, such as a pain signal [19–21]. This phenomenon is particularly noteworthy in the tumor microenvironment. TN breast cancer tissues commonly exhibit increased mechanical stress and elevated stromal stiffness [22, 23]. This abnormality in the biomechanical microenvironment may directly contribute to the occurrence and amplification of local pain. Therefore, the intrinsic link between mechanical stimulation and pain provides an important entry point for understanding the mechanisms of breast cancer pain.

The treatment of tumors is inseparable from the microenvironment upon which they rely for survival. Fibroblasts are the most abundant and functionally active type of mesenchymal cell [24, 25]. With the development of single‐cell technology, researchers have gradually realized that fibroblasts are not a single cell population, but rather exhibit significant functional heterogeneity, meaning that different fibroblasts show significant differences in gene expression, phenotypic characteristics, and functions [26]. Existing studies have confirmed that multiple fibroblast subtypes exist in TN breast cancer, and they play different or even opposite roles in tumor growth, invasion and metastasis, and immune regulation [27–29]. This heterogeneity provides a new perspective for understanding the multiple functions of fibroblasts in tumor biology and suggests the possible existence of specific functional subtypes in pain regulation. Meanwhile, fibroblasts are key regulators of tissue mechanical properties; by remodeling the extracellular matrix, fibroblasts directly determine the stiffness and mechanical stress levels of local tissues [30–33]. Based on this function of fibroblasts in regulating mechanical properties, and combined with the intrinsic link between mechanical stimulation and pain, it can be inferred that specific fibroblast subtypes may participate in the occurrence of breast cancer pain by altering the local mechanical microenvironment [34].

However, the current understanding of the specific role of fibroblasts in TN breast cancer pain remains at the correlation level. There is a lack of systematic single‐cell‐level analysis regarding whether specific mechanical stress–related fibroblast subtypes can influence pain intensity by altering the local biomechanical microenvironment. Therefore, this study selected the publicly available single‐cell RNA sequencing (scRNA‐seq) dataset, which contains single‐cell transcriptome data from TN breast cancer tissue and healthy control (HC) samples, aiming to conduct a systematic analysis of the tumor microenvironment. This study first characterized the compositional changes of major cell types in the tumor microenvironment at a holistic level and systematically explored the functional enrichment and mechanical scores of different fibroblast subtypes. Integrating information on biomechanical abnormalities, fibroblast heterogeneity, and clinical symptoms, this study aims to reveal the potential regulatory mechanisms of TN breast cancer pain and provide experimental evidence for the development of related risk assessment and intervention strategies.

## 2. Materials and Methods

### 2.1. Single‐Cell Data Processing

The scRNA‐seq data used in this study were obtained from the Gene Expression Omnibus (GEO) (https://www.ncbi.nlm.nih.gov/geo/), with accession number: GSE161529. This dataset contains single‐cell transcriptome data from TN breast cancer tissue and HC samples, covering 71,293 cells.

Analysis was performed using the Seurat R package (V4.3.0). Data quality control was performed using the DoubletFinder R package (V2.0.3). The quality control indicators are as follows: (1) 300 < nFeature < 6000, (2) 500 < nCount < 100,000, (3) mitochondrial gene expression does not exceed 25% of the total number of genes in the cell, and (4) erythroid gene expression does not exceed 5% of the total number of genes in the cell. For high‐quality single cells retained after quality control, logarithmic normalization was performed using the “NormalizeData” function in the Seurat R package (V4.3.0). Subsequently, highly variable genes were screened using the “FindVariableFeatures” function, and data standardization was performed using the “ScaleData” function. Principal component analysis was performed on the 2000 genes with the highest variability, and the Harmony algorithm (V0.1.1) was used to correct for the influence of different sample batches [35–37]. The first 30 principal components were selected for further analysis. The dim value was set to 30 and the resolution to 1.2.

### 2.2. Cell Type Identification and Subtype Analysis

Based on the CellMarker database (https://xteam.xbio.top/CellMarker/) and canonical markers for cell subtypes—and utilizing the automatic annotation functionality of “SingleR”—preliminary identification of cell clusters was performed using the “FindClusters” and “FindNeighbors” functions implemented in the Seurat software [38, 39]. The marker genes used to define each fibroblast subtype were selected based on differential expression analysis, with careful consideration given to both statistical significance and biological interpretability.

### 2.3. Functional Enrichment Analysis

The Seurat “Find All Markers” function was used to conduct the Wilcoxon rank‐sum test for the assessment of differentially expressed genes (DEGs) across various cellular clusters [40–44]. The min.pct and min.diff.pct were set to 0.25, and the logfc threshold was set to 0.25. Enrichment analyses of DEGs in various cell types were conducted using Gene Ontology (GO) and Gene Set Enrichment Analysis (GSEA) (https://software.broadinstitute.org/gsea/msigdb) tools using the clusterProfiler R package (v4.14.0). The threshold is |logFC|> 2 with *p* value less than 0.05.

### 2.4. Single‐Cell Metabolic Pathway Activity Analysis

The scMetabolism R package (V0.2.1) was used to quantitatively assess single‐cell metabolic activity based on the metabolic pathway gene set in the Kyoto Encyclopedia of Genes and Genomes database. This method integrates gene expression data with metabolic network topology to calculate the relative activity score of each cell in each metabolic pathway [45, 46].

### 2.5. Area Under the Curve Cell (AUCell) Rating

This study used the scgmt R package (V0.0.3, https://github.com/ZhaoLabs-SJTU/scgmt) to score the activity of mechanical signals and pain gene sets and selected the AUCell method (V1.20.2) as the core analysis tool [47]. These gene sets were primarily sourced from the MSigDB website (https://www.gsea-msigdb.org) and previous literature [48]. For specific details, please refer to Supporting Table 1. The Wilcoxon test was used for comparison between two groups, and the Kruskal test was used for comparison between multiple groups.

### 2.6. Cell‐To‐Cell Communication Analysis

Based on the dataset derived from all sample sources, this study utilized the CellChat R package (V1.6.1) to infer the levels of intercellular ligand–receptor interactions and constructed the corresponding regulatory networks. We used heatmaps to illustrate the signal input and output characteristics of each cell type and focused on analyzing the communication relationships and related signaling pathways between fibroblasts and other cell types [49, 50]. We utilized a significance threshold setting with a *p* value cutoff of 0.05 to predict cell–cell interactions between different cell types.

### 2.7. SCENIC Analysis

To identify transcription factors in fibroblasts, we used the SCENIC package (V1.3.1) in Python 3.7 to perform single‐cell regulatory network inference and cluster analysis [51]. Initially, GRNBoost was utilized to pinpoint prospective target genes for each transcription factor. After that, DNA‐motif analysis helped identify potential direct binding targets. The activity of the regulon in the cells was assessed using AUCell, and the top five transcription factors with the highest scores were identified.

### 2.8. Statistical Analysis

R software and Python software are used for database data analysis. All mentioned *p* values are two‐tailed, with values below 0.05 considered statistically significant. Values below 0.001 were deemed highly significant, while those below 0.0001 were considered extremely significant [52–54].

## 3. Results

### 3.1. Single‐Cell Transcriptomics Analysis Reveals Cellular Heterogeneity in TN Breast Cancer

We clustered and classified TN breast cancer tissue cells into seven categories: myeloid cells, B cells, plasma cells, endothelial cells, stromal cells, T‐NK cells, and epithelial cells (Figure 1A). Excluding endothelial and stromal cells, which mostly came from the HC group, the other six cell types were primarily derived from the TN group, with the majority in the G1 phase. Notably, immune cell infiltration was markedly lower in normal breast tissue. Figure 1B illustrates the criteria used for cell type classification. Compared to other cell types, epithelial cells exhibited higher scores for the G2/M and S phases of the cell cycle, suggesting active cellular proliferation (Figures 1C and D). This observation is also consistent with the Ro/e results (Figures 1E and F). A visualization analysis of DEGs across these seven cell types, utilizing a volcano plot, revealed that upregulated genes in stromal cells included FTL, RASSF8, GNG11, ECM1, and TNS1 (Figure 1G). These genes were primarily enriched in GO biological processes such as cytoplasmic translation, positive regulation of cytokine production, and positive regulation of response to external stimuli (Figure 1H). GSEA results similarly indicated a positive enrichment trend in stromal cells regarding pathways related to collagen fibril assembly and the extracellular matrix (Figure 1I).

FIGURE 1Comprehensive cellular landscape and functional characteristics of single‐cell transcriptomes (A) UMAP plot illustrating the clustering distribution of all cells, encompassing epithelial cells, endothelial cells, stromal cells, myeloid cells, T‐NK cells, B cells, and plasma cells. The legend on the right is color‐coded according to grouping (HC and TN) and cell cycle phase (G1, S, and G2/M). (B) Marker genes for different cell types. (C–D) Distribution and expression levels of nCount RNA (total RNA counts), nFeature RNA (number of detected genes), G2/M score, and S score. (E–F) Ro/e scores for each cell type across the HC and TN groups, as well as across the G1, S, and G2/M cell phases. (G) Volcano plots displaying DEGs within each cell type. (H) Bar plot of biological processes (GO terms) identified through enrichment analysis. (I) GSEA results for each cell type.

**Figure:**
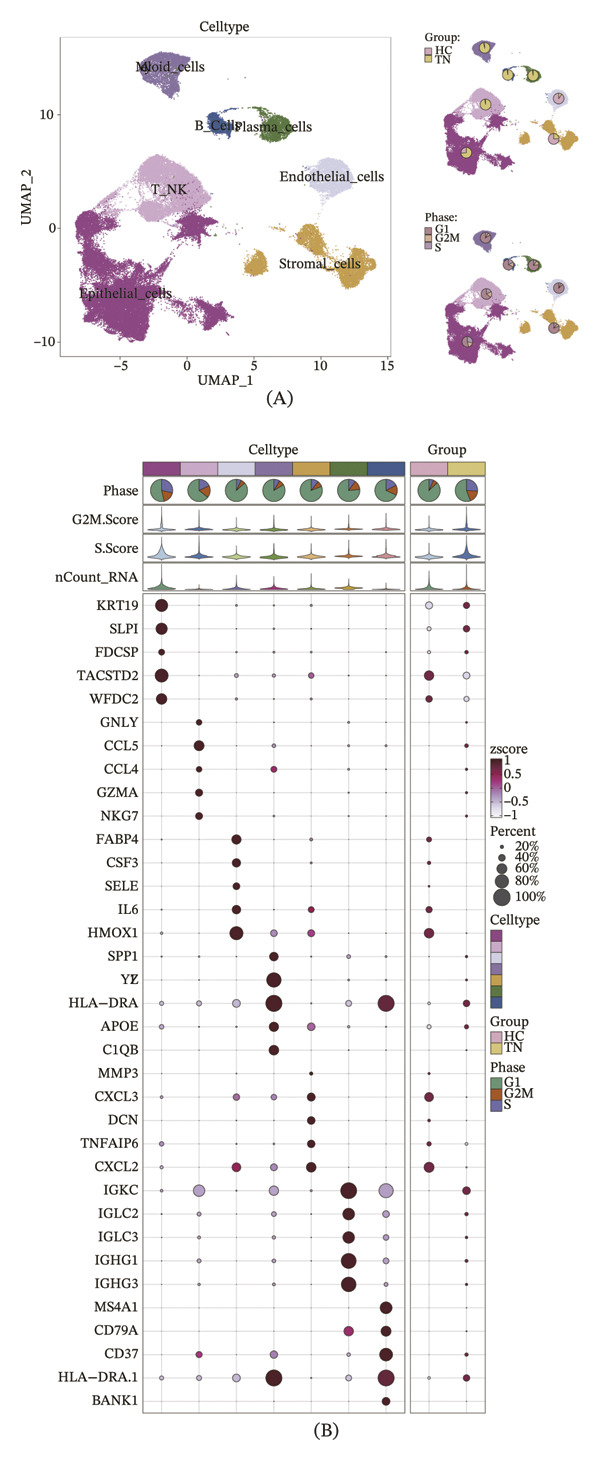

**Figure:**
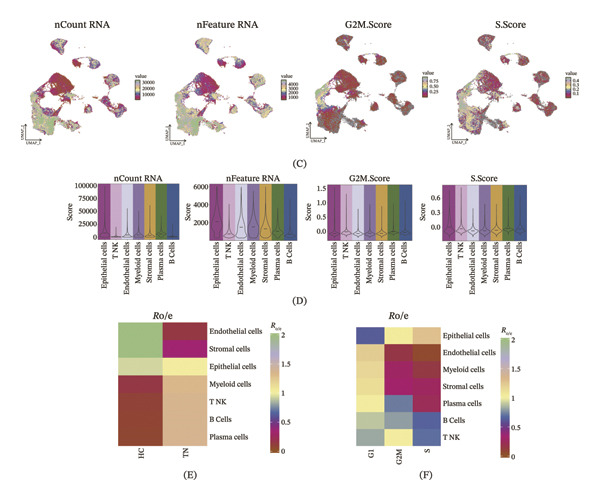

**Figure:**
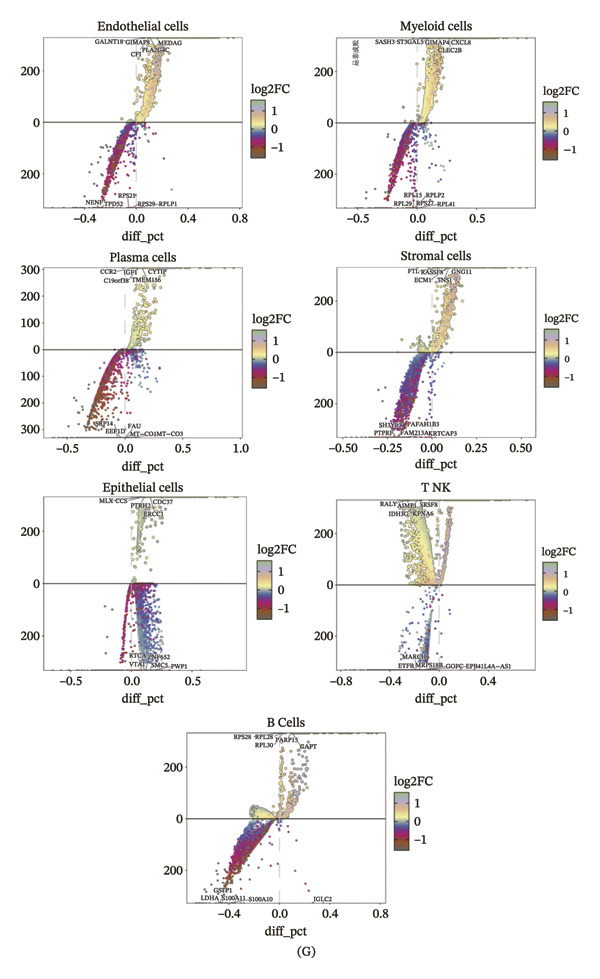

**Figure:**
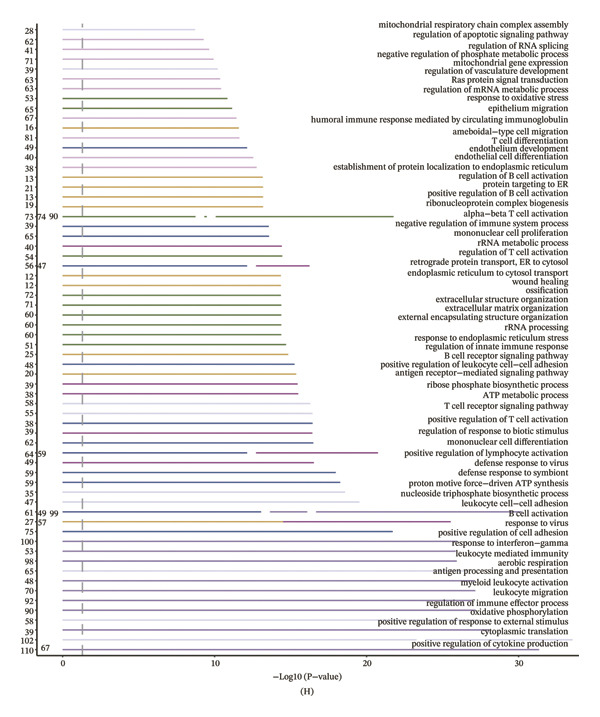

**Figure:**
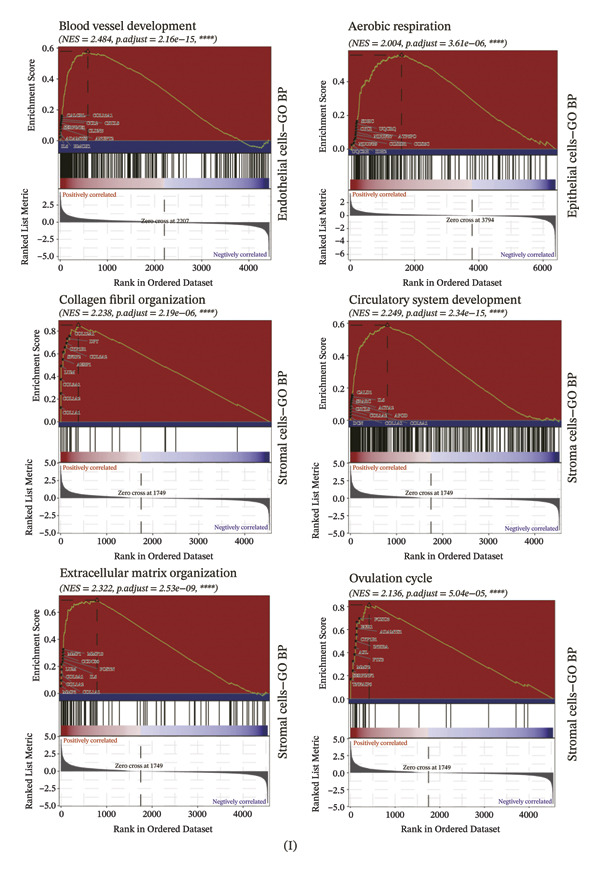

### 3.2. Single‐Cell Biological Characteristics of Stromal Cells in TN Breast Cancer

**FIGURE 2:**
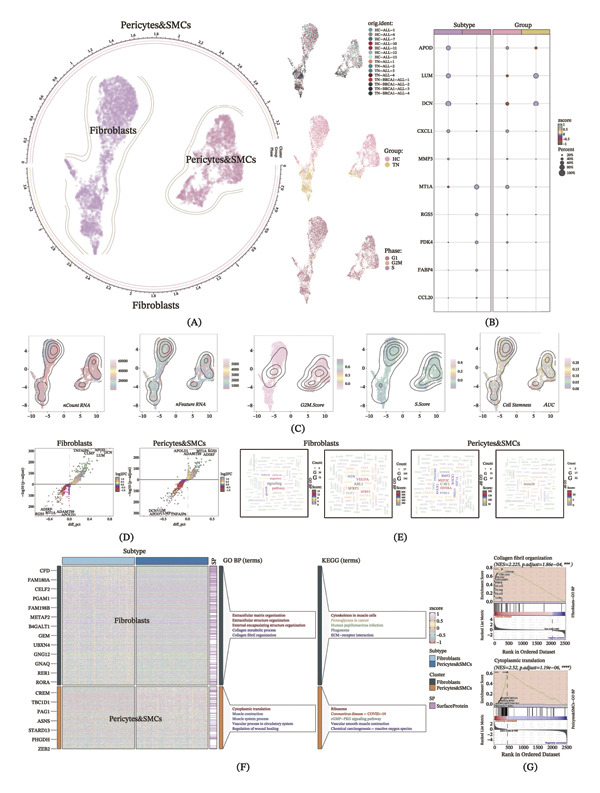
Single‐cell landscape of stromal cells (A) UMAP plots of fibroblasts and pericytes/smooth muscle cells, visualized by cell origin (cell type), sample group (TN and HC), and cell cycle phase (G1, G2/M, and S). (B) Bubble plot displaying marker genes for fibroblasts and pericytes/smooth muscle cells. (C) Contour UMAP plots illustrating the distribution of nCount RNA, nFeature RNA, G2/M score, S score, and cell stemness AUC across various cell subtypes. (D) Volcano plot displaying DEGs in fibroblasts and pericytes/smooth muscle cells. (E) Word cloud visualizing the functional activity scores of fibroblasts and pericytes/smooth muscle cells. (F–G) Heterogeneity in biological processes between fibroblasts and pericytes/smooth muscle cells.

Next, we further analyzed and clustered stromal cells, identifying three cell types: fibroblasts, pericytes, and smooth muscle cells (SMCs) (Figure 2A). The UMAP plot illustrates the distribution of sample origins, groupings, and cell cycle phases across these three cell types. Notably, even after rigorous quality control measures were applied to eliminate batch effects, the UMAP plot still revealed significant differences among the three stromal cell types derived from normal tissues versus tumor tissues. A bubble plot displays the differentially expressed marker genes across the various cell types and groupings (Figure 2B). As shown in Figure 2C, UMAP contour plots further provide a visual representation of the expression distributions for nCount RNA, nFeature RNA, G2/M scores, S scores, and cell stemness AUC values. Enrichment analysis of the DEGs (Figure 2D) indicated that fibroblasts are primarily enriched in functions related to the extracellular matrix, collagen metabolism, and collagen fibers (Figures 2E and G). Among these, the extracellular matrix serves not only as a physical scaffold for cells but also as a critical mediator through which mechanical stimuli regulate cellular behavior. Consequently, we hypothesize that fibroblasts may influence the pathophysiological progression and clinical manifestations of breast cancer by altering their mechanical properties.

### 3.3. Transcriptional Heterogeneity and Functional State Differences of Fibroblast Subtypes in TN Breast Cancer

**FIGURE 3:**
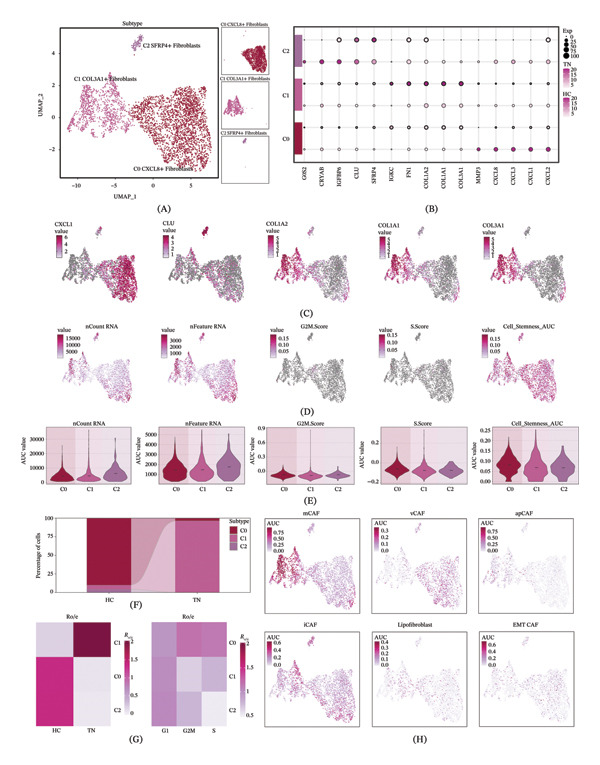
Identification, functional characterization, and tissue specificity analysis of fibroblast subtypes. (A) UMAP plot illustrating the distribution of three major fibroblast subtypes identified through dimensionality reduction and clustering. (B) Enrichment bubble plot displaying the top 5 marker genes for each of the three fibroblast subtypes. (C) UMAP plot visualizing the expression levels of five marker genes (CXCL1, CLU, COL1A2, COL1A1, and COL3A1) within the fibroblast population. (D–E) UMAP plots and violin plots illustrating the distribution of nCount RNA, nFeature RNA, G2/M scores, S scores, and cell stemness AUC values across all fibroblast subtypes in both groups. The Wilcoxon test was used for comparison between two groups, and the Kruskal test was used for comparison between multiple groups. (F–G) Stacked bar charts depicting the proportional distribution and tissue specificity of fibroblast subtypes across different tissue origins. (H) Six UMAP plots visualizing the distribution of AUC scores for CAF‐associated functional features across the various fibroblast subtypes.

To further investigate the mechanical properties of these fibroblasts, we classified them into three subtypes based on differentially expressed marker genes: C0 CXCL8^+^ fibroblasts, C1 COL3A1^+^ fibroblasts, and C2 SFRP4^+^ fibroblasts (Figures 3A and B). A UMAP dimensionality reduction plot was used to visualize the marker genes highly expressed within each fibroblast subtype (Figure 3C). Furthermore, C2 SFRP4^+^ fibroblasts demonstrated higher scores for the nCount RNA and nFeature RNA metrics (Figures 3D and E), while C0 CXCL8^+^ fibroblasts exhibited more prominent proliferative characteristics. Notably, C1 COL3A1^+^ fibroblasts were predominantly derived from the TN breast cancer group, whereas the C0 and C2 subtypes were primarily derived from the HC group (Figures 3F and G). This suggests that the functional characteristics of fibroblasts exhibit significant heterogeneity within tumor tissues. Consequently, we performed AUCell scoring on the fibroblasts in this study, utilizing the functional signatures of six canonical cancer‐associated fibroblasts (CAFs) (Figure 3H). The results revealed that the stromal and immune signatures of C1 COL3A1^+^ fibroblasts were significantly enhanced. Notably, previous studies have found that matrix CAFs are primarily enriched in processes related to extracellular matrix remodeling, tend to be localized at the tumor–stroma interface, and can assist tumor cells in forming a dense stromal barrier [55]. This aligns with the characteristics exhibited by the C1 COL3A1^+^ fibroblasts in the present study. However, it must be emphasized that this consistency is based solely on similarities at the transcriptomic level and requires further experimental validation. In any event, the aforementioned results suggest that, under the influence of the tumor microenvironment, fibroblasts within TN breast cancer may possess enhanced stromal remodeling capabilities.

### 3.4. C1 COL3A1^+^ Fibroblasts Exhibit High Expression of Mechanostimulation‐Related Genes

Next, we further dissected the biological heterogeneity of C1 COL3A1^+^ fibroblasts. The differentially upregulated genes in C1 COL3A1^+^ fibroblasts included IFITM3, CALD1, COL1A2, COL5A2, and COL3A1 (Figure 4A). GO functional activity scoring of these DEGs revealed that C1 COL3A1^+^ fibroblasts exhibited higher scores for functions related to actin, stimulation, and morphogenesis (Figure 4B). GO pathway enrichment and GSEA results further confirmed that C1 COL3A1^+^ fibroblasts displayed positive enrichment in functions associated with collagen fibrils, extracellular structures, and the extracellular matrix (Figures 4C and D). Concurrently, we calculated the AUCell scores for various actin pathway genes across each fibroblast subtype. Consistent with the results of the enrichment analysis, the C1 COL3A1^+^ fibroblasts exhibited high expression of genes associated with the actin cytoskeleton, as well as actin filament motility and polymerization, while the expression levels of microtubule‐related genes remained relatively low (Figure 4E). To investigate the heterogeneity of mechanical stimuli within the C1 COL3A1^+^ fibroblasts and to systematically assess the potential transcriptomic links between these mechanical stimuli and pain, we also calculated the AUCell scores for genes involved in mechanical stimulation and pain pathways across each fibroblast subtype. The results demonstrated that genes associated with both mechanical stimulation and pain were significantly upregulated in the C1 COL3A1^+^ fibroblasts (Figure 4F). To better validate the expression of genes associated with mechanical stimulation and pain response within this fibroblast subtype at the clinical level, we first employed Venn diagram analysis to identify the intersection of the top four mechanical stimulation–related gene sets shown in Figure 4F, ultimately identifying three characteristic mechanical stimulation genes: CTNNB1, FYN, and PECAM1. Based on the differential expression patterns and biological interpretability of these three genes, we selected CTNNB1 for subsequent validation (Figure 4G). Similarly, we performed Venn diagram analysis on the two pain‐related gene sets presented in Figure 4F and selected TRPA1 for subsequent validation (Figure 4H). As illustrated in Figure 4I, we conducted immunohistochemical analysis on tumor tissue samples from patients with or without cancer‐related pain to assess the expression levels of the fibroblast marker COL3A1, the mechanical signaling marker CTNNB1, and the pain marker TRPA1. We observed that, in patients experiencing cancer‐related pain, the expression levels of all three markers consistently demonstrated an upregulation. This suggests that high expression of COL3A1 in fibroblasts may play a potential role in pain sensitivity in cancer patients.

FIGURE 4Biological functional characteristics of fibroblast subtypes. (A) Volcano plot showing DEGs in fibroblast subtypes. (B) Word cloud plot showing functional activity of different fibroblast subtypes. (C) Bar plot showing GO pathway enrichment in each fibroblast subtypes. (D) GSEA results for different pathways in fibroblast subtypes. (E) Violin plot showing the distribution of cytoskeleton‐related pathway activity scores across all fibroblast subtypes in both groups. The Wilcoxon test was used for comparison between two groups, and the Kruskal test was used for comparison between multiple groups. (F) Violin plot and UMAP contour plot showing the scores and distribution of six gene sets related to mechanosensory and pain response from the GSEA database across all fibroblast subtypes in both groups. Specific gene sets include sensory perception of mechanical stimulus, detection of mechanical stimulus, response to mechanical stimulus, cellular responses to mechanical stimuli, mechanical stimulus involved in sensory perception of pain; and response to pain. The Wilcoxon test was used for comparison between two groups, and the Kruskal test was used for comparison between multiple groups. (G–H) The heatmap illustrated the intersection of mechanosensory gene sets (G) and pain‐response gene sets (H), as well as the expression levels of individual genes within these shared signatures. The bar plots displayed the expression levels of the mechanosensory gene CTNNB1 and the pain‐related gene TRPA1. The Wilcoxon test was used for comparison between two groups, and the Kruskal test was used for comparison between multiple groups. (I) Immunohistochemical images of COL3A1, CTNNB1, and TRPA1 in patients with cancer pain and noncancer pain.

**Figure:**
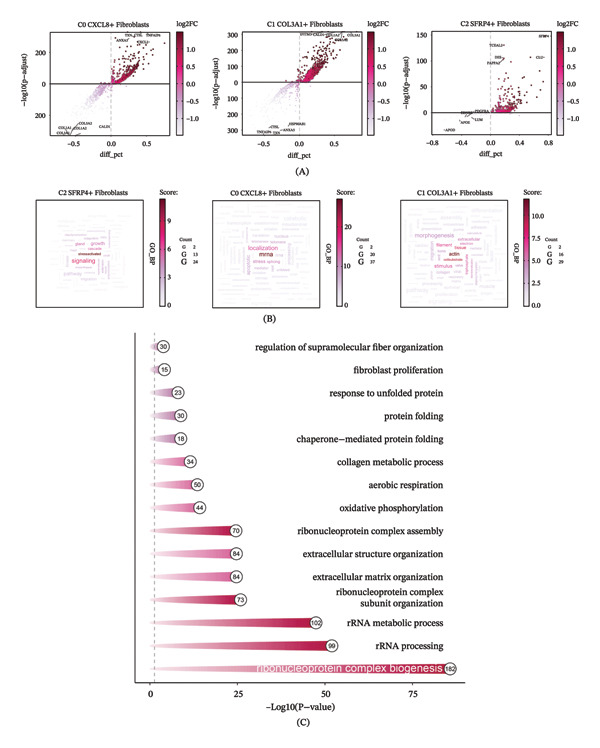

**Figure:**
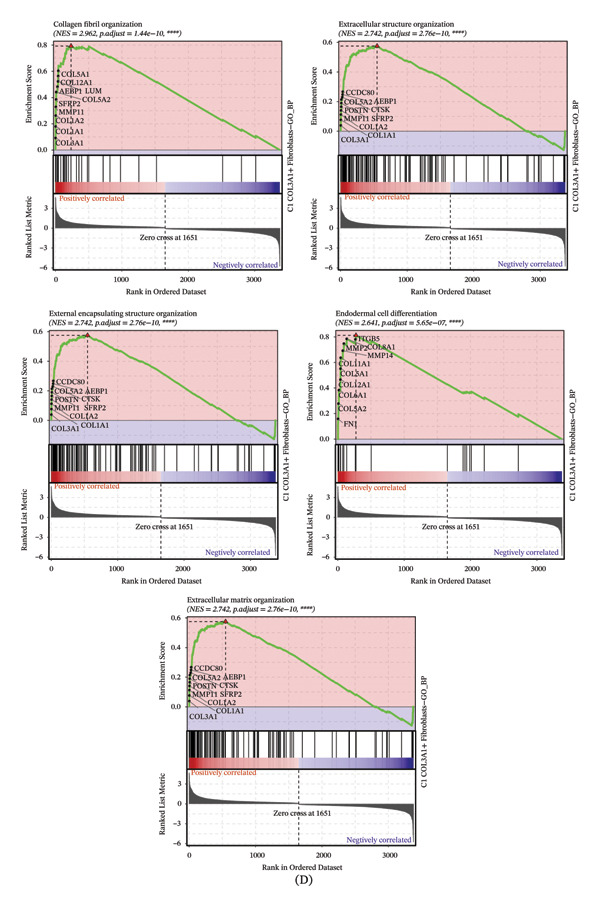

**Figure:**
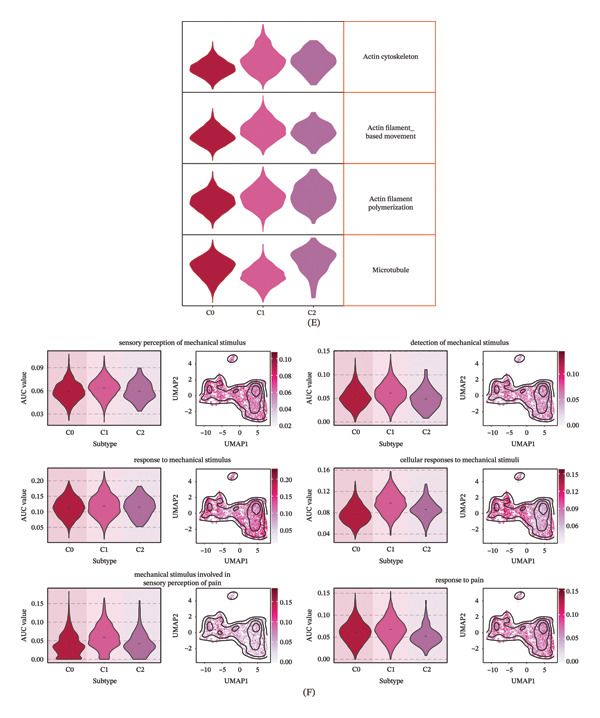

**Figure:**
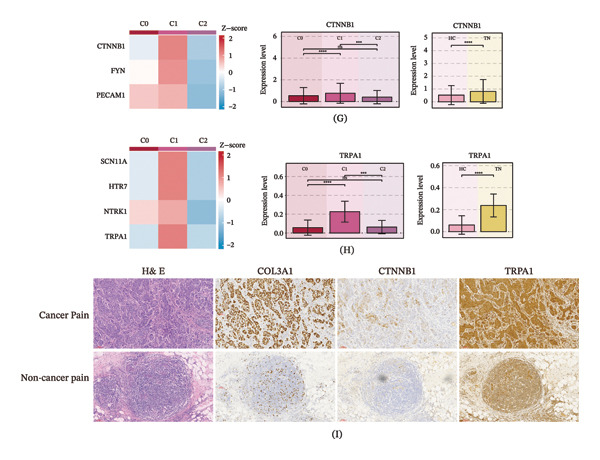

### 3.5. Laminin and Collagen Signaling Pathways Are Significantly Active in C1 COL3A1^+^ Fibroblasts

To investigate the potential mechanosignaling networks within C1 COL3A1^+^ fibroblasts, we utilized intercellular communication analysis to infer the intricate communication networks existing between fibroblasts and other cell types (Figure 5A). The analysis revealed that C1 COL3A1^+^ fibroblasts demonstrated exceptional performance in terms of both the total number and intensity of interactions with other cells; notably, they appear to engage in particularly strong interactions—at the transcriptomic level—with epithelial cells and myeloid cells (Figure 5B). Figure 5C illustrates the expression profiles of all outgoing and incoming signals across the various cell types. C1 COL3A1^+^ fibroblasts primarily participate in signaling pathways such as laminin, collagen, FN1, and ITGB. Significantly, all these pathways are associated with the extracellular matrix and mechanotransduction. Within the laminin pathway, C1 COL3A1^+^ fibroblasts act as the primary signal senders, exhibiting communication intensities with endothelial and epithelial cells that are significantly higher than those observed with other cell types. Similarly, in the collagen pathway, C1 COL3A1^+^ fibroblasts also demonstrated the strongest signal‐sending capabilities, engaging in significant interactions with endothelial cells (Figure 5D). Ligand–receptor expression analysis inferred that C1 COL3A1^+^ fibroblasts express key molecules—including ITGB1, ITGA2, LAMA4, LAMB1, COL1A1, and COL6A3—at high levels; this likely provides the molecular basis for their pivotal roles within the aforementioned signaling pathways (Figure 5E). In both the laminin and collagen pathways, C1 COL3A1^+^ fibroblasts appear to simultaneously fulfill the triple roles of signal sender, mediator, and influencer, evidenced by their significantly higher importance scores compared to other cell subtypes. This suggests that they not only actively transmit signals but also play a critical hub‐like role and exert a signal‐amplifying effect throughout the entire signaling network (Figure 5F). Global network visualization results suggest that C1 COL3A1^+^ fibroblasts may serve as key nodes connecting endothelial cells, epithelial cells, and other immune cells (Figure 5G). It should be noted, however, that while the COL3A1^+^ fibroblasts within the C1 subtype may be associated—at the transcriptomic level—with enhanced laminin and collagen signaling interactions, their core regulatory role has not yet been definitively confirmed.

FIGURE 5Intercellular communication networks and key signaling pathways mediated by fibroblast subtypes. (A) Circular plot showing the number (left) and intensity (right) of interactions between fibroblasts and other cell types. (B) Circular plot showing the number (left) and intensity (right) of intercellular interactions with C1 fibroblasts as signaling sources or target cells. (C) Heatmap showing afferent and efferent signaling patterns in various cell types across different pathways. (D) Chord and hierarchical plots visualizing the laminin (top) and collagen (bottom) signaling networks, showing communication relationships between fibroblast subtypes and target cells. (E) Dot plot showing the expression patterns of key ligand–receptor molecules in the laminin (left) and collagen (right) pathways across different cell types. (F) Heatmap showing the importance scores of fibroblast subtypes as senders, receivers, mediators, and influencers in the laminin (left) and collagen (right) pathways. (G) The circular diagram illustrates the interaction of fibroblast subtypes with other cells in the laminin (left) and collagen (right) signaling pathways.

**Figure:**
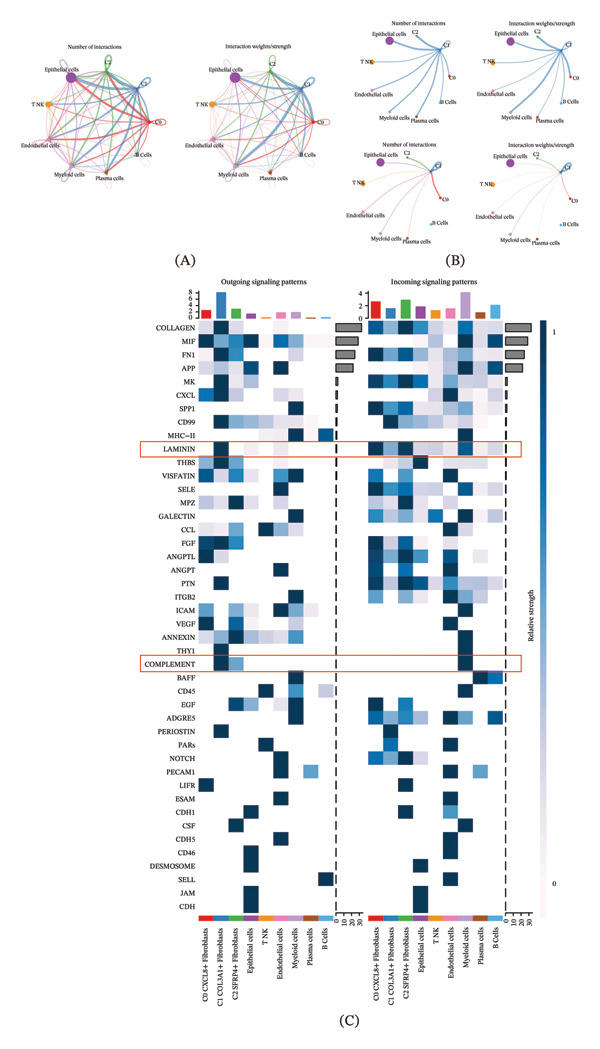

**Figure:**
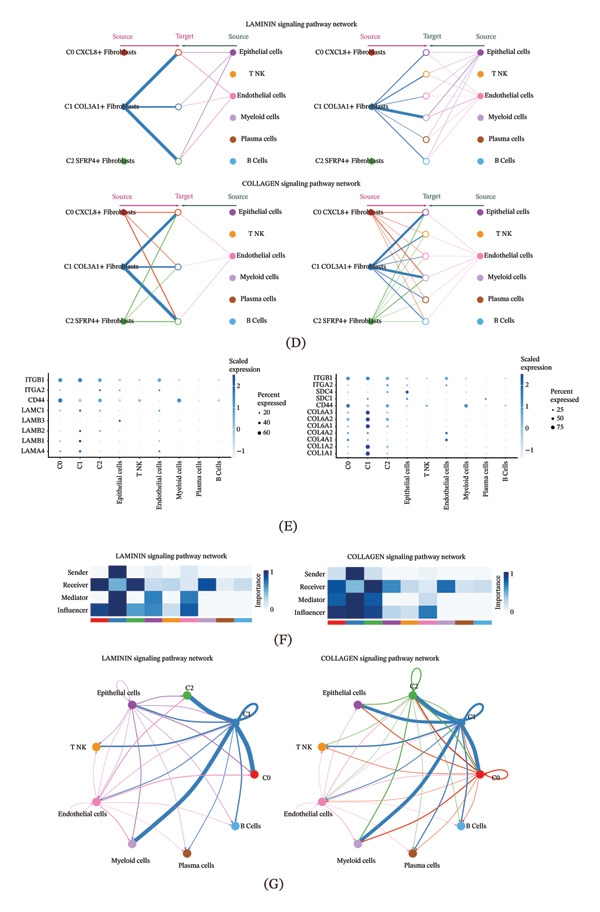

### 3.6. Transcriptional Regulatory Network of C1 COL3A1^+^ Fibroblasts

**FIGURE 6:**
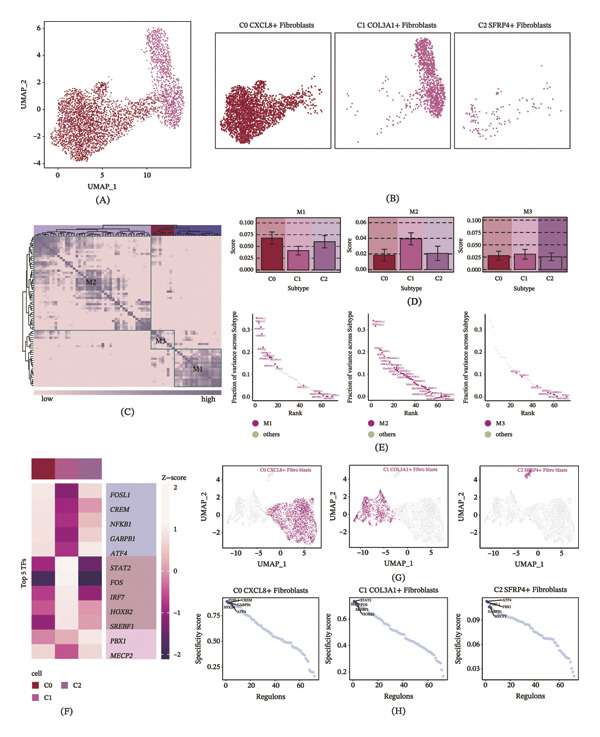
Transcriptional regulatory characteristics of fibroblasts (A–B) UMAP plots showing the re‐clustering of fibroblasts based on differential expression of transcription factors. (C) Three transcription factor regulatory modules of fibroblast subtypes. (D) Activity scores of the three transcription factor regulatory modules in different fibroblast subtypes. (E) Variance contribution of each transcription factor regulatory module to the heterogeneity of fibroblast subtypes. (F–H) Expression of the top 5 characteristic transcription factors in fibroblast subtypes.

To investigate the regulatory mechanisms underlying the mechanical signaling heterogeneity of C1 COL3A1^+^ fibroblasts, we utilized SCENIC to conduct an exploratory analysis of their potential transcriptional regulatory networks. UMAP visualization results revealed a distinct separation of the three fibroblast subtypes within the dimensionality‐reduced space, suggesting that they may possess significant differences in their transcriptional profiles (Figures 6A and B). Clustering analysis based on transcription factor expression profiles identified three major functional modules—M1, M2, and M3—each exhibiting a unique pattern of activity across the different subtypes (Figures 6C and D). Notably, the M2 module demonstrated the highest level of activity within the C1 COL3A1^+^ fibroblasts. The results of the analysis of variance indicate that transcriptional differences between Modules M1 and M2 may be the underlying drivers of the aforementioned heterogeneity observed among fibroblast subtypes (Figure 6E). Furthermore, compared to other fibroblast subtypes, C1 COL3A1+ fibroblasts exhibit higher expression of STAT2, IRF7, FOS, SREBF1, and HOXB2 (Figures 6F and H)—factors closely associated with extracellular matrix synthesis and angiogenesis, for which experimental validation is still required.

## 4. Discussion

This study systematically analyzed the biological functional characteristics of fibroblasts in TN breast cancer and further explored the potential transcriptomic link between the tumor’s mechanical microenvironment and the onset of pain. Pathways associated with the extracellular matrix, extracellular structures, and collagen metabolism were found to be significantly activated within the fibroblasts of tumor tissues; this finding preliminarily suggests that these cells may play a role in altering the biomechanical microenvironment of breast cancer tissues [56, 57]. The tumor microenvironment of breast cancer exhibited significant stromal remodeling characteristics, manifested as increased tissue stiffness. This stiffness change originated from the excessive secretion of extracellular matrix components such as collagen by fibroblasts, forming a dense, desmoplastic matrix [58]. When the matrix stiffness exceeded the level of normal tissue, it would activate intracellular mechanotransduction signals through the integrin‐focal adhesion pathway, causing fibroblasts to be in a state of continuous tension activation [59]. This alteration of the mechanical microenvironment not only affected tumor cell behavior but might also play a key role in the development of tumor pain by activating mechanosensitive channels in sensory nerve endings and promoting neurosensitization [60].

Fibroblasts were the main source of extracellular matrix, and they drove desmoplasia. High‐rigidity tissue promoted nerve sensitization, leading to pain [61–63]. C1 COL3A1^+^ fibroblasts showed the highest proportion and Ro/e score in the TN breast cancer, suggesting selective enrichment in tumor tissue. C1 COL3A1^+^ fibroblasts were characterized by high expression levels of matrix synthesis–related genes, including collagen genes such as COL1A1, COL1A2, COL3A1, and COL5A2, as well as FN1. This high expression directly led to the secretion of large amounts of extracellular matrix proteins by this subtype, which was the molecular basis for increased matrix deposition and tissue stiffness [56]. GSEA analysis further validated that C1 COL3A1^+^ fibroblasts are upregulated in the pathways of collagen fibril organization, extracellular matrix organization, external encapsulating structure organization, and endodermal cell differentiation, and their function in collagen fiber formation and structural remodeling was enhanced. C1 COL3A1^+^ fibroblasts are active in biological processes such as actin, cell substrate, tissue, filament, stimulation, and morphogenesis, suggesting that they may be involved in a cellular state characterized by high tension and heightened sensitivity to mechanical stimuli.

The development of tumor pain involved three intertwined pathways: direct nerve compression or infiltration, stimulation by inflammatory mediators, and activation by mechanical factors [59]. This study focuses on investigating the potential role that mechanical factors may play in the process of pain induction. By constructing a set of gene signatures for scoring mechanosensation and pain response, the results suggest an overlap in the expression of genes characteristic of mechanical stress and those characteristic of pain; this implies a potential link between fibroblast‐mediated alterations in the tumor mechanical microenvironment and the molecular mechanisms underlying the onset of pain. This conclusion is also consistent with previous studies [64, 65].

AUCell [66] analysis showed that C1 COL3A1^+^ fibroblasts exhibited the highest enrichment scores in mechanosensitive pathways, including responses to mechanical stimuli, cellular responses to mechanical stimuli, mechanosensory perception, and pain‐related pathways. This pattern suggests that this subtype may not merely be a passive producer of the extracellular matrix, but rather a potentially active cell type capable of sensing and responding to mechanical stimuli. Notably, this aligns with the functional definition of “mechanosensitive fibroblasts” described in previous studies [67, 68].

Based on evidence from previous studies and the findings of the current study, we hypothesize that C1 COL3A1^+^ fibroblasts may function as a progenitor‐like cell population. By continuously producing extracellular matrix, they maintain a state of high stromal stiffness during tumor progression, thereby serving as a persistent source of stimulation for mechanically induced pain [69, 70]. Cell–cell communication analysis suggests that C1 COL3A1^+^ fibroblasts may play a pivotal role in both laminin and collagen signaling pathways, acting as signal senders, mediators, and influencers. Previous studies have found that increased collagen deposition enhances tissue stiffness, while integrin‐mediated mechanotransduction further augments both cytoskeletal contractility and matrix remodeling capabilities; this process continuously reinforces the mechanical microenvironment and promotes the sustained activation of pain‐related transcriptional programs [64]. Given that the transcriptional regulatory programs of C1 COL3A1^+^ fibroblasts are skewed toward matrix production and structural remodeling, our findings may provide single‐cell‐level support for a potential link between matrix remodeling and pain hypersensitivity.

This study had prospective significance in the field of breast cancer pain. At the predictive level, COL3A1^+^ fibroblasts could serve as a biomarker for potential pain risk. These findings may provide preliminary insights into potential biological processes associated with tumor‐associated pain; however, their therapeutic implications remain speculative and require further experimental and clinical validation. Furthermore, the study lacked direct clinical pain scoring data, spatial localization validation, and functional experimental validation. These findings provided hypothesis‐generating evidence rather than definitive mechanistic proof. Future research would focus on spatial transcriptomics, organoid models, and in vivo mechanical modulation models, paving the way for precision biomedical delivery systems and drug delivery therapy [71–80].

## 5. Conclusion

This study reveals the existence of a stromal cell subtype within breast cancer tissue—centered around COL3A1^+^ fibroblasts—that is associated with mechanical activation and pain sensitivity. This subtype may contribute to the process of tissue stiffening by increasing collagen deposition, remodeling the cytoskeleton, and amplifying mechanotransduction signals. Furthermore, it may be linked to mechanosensitive neural pathways, participating in the activation of pain‐related molecular programs. At the single‐cell level, this study proposes a potential underlying coupling mechanism—involving “stromal stiffness, mechanical stress, and neural sensitization”—that may drive breast cancer‐associated pain, thereby offering new insights for the stratified management and therapeutic strategies of cancer‐related pain.

## Author Contributions

Yuting Zhong, Qi Sun, and Changgang Sun conceived and designed the research. Yuting Zhong was responsible for manuscript preparation and writing. Qi Sun was responsible for data collection and the analysis of single‐cell RNA sequencing. Changgang Sun provided funding support and supervision. Yuting Zhong and Qi Sun are co‐first authors.

## Funding

This research was supported by the National Natural Science Foundation of China Key Program (82430123), the Shandong Provincial Taishan Scholars Distinguished Expert Program (tstp20221166), and the National Administration of Traditional Chinese Medicine High‐Level Key Discipline Construction Project (ZYYZDXK‐2023125).

## Disclosure

All authors have read and approved the final manuscript.

## Ethics Statement

All tissue specimens were provided by the Weifang Hospital of Traditional Chinese Medicine. Written informed consent was obtained from each participant prior to sample collection. The study protocol was reviewed and approved by the Ethics Review Committee of the Weifang Hospital of Traditional Chinese Medicine (Approval No.: 2023YX031).

## Consent

Please see the Ethics Statement.

## Conflicts of Interest

The authors declare no conflicts of interest.

## Supporting Information

Additional supporting information can be found online in the Supporting Information section.

## Supporting information


**Supporting Information** Supporting Materials. 1 Supporting Table 1. List of related gene sets.

## Data Availability

The single‐cell RNA sequencing data analyzed in this study are publicly available in the GEO database under accession number: GSE161529. All data used in this study can be accessed without restriction.
